# Shifting suitability for malaria vectors across Africa with warming climates

**DOI:** 10.1186/1471-2334-9-59

**Published:** 2009-05-10

**Authors:** A Townsend Peterson

**Affiliations:** 1 Biodiversity Research Center, The University of Kansas, Lawrence, Kansas 66045, USA

## Abstract

**Background:**

Climates are changing rapidly, producing warm climate conditions globally not previously observed in modern history. Malaria is of great concern as a cause of human mortality and morbidity, particularly across Africa, thanks in large part to the presence there of a particularly competent suite of mosquito vector species.

**Methods:**

I derive spatially explicit estimates of human populations living in regions newly suitable climatically for populations of two key *Anopheles gambiae *vector complex species in Africa over the coming 50 years, based on ecological niche model projections over two global climate models, two scenarios of climate change, and detailed spatial summaries of human population distributions.

**Results:**

For both species, under all scenarios, given the changing spatial distribution of appropriate conditions and the current population distribution, the models predict a reduction of 11.3–30.2% in the percentage of the overall population living in areas climatically suitable for these vector species in coming decades, but reductions and increases are focused in different regions: malaria vector suitability is likely to decrease in West Africa, but increase in eastern and southern Africa.

**Conclusion:**

Climate change effects on African malaria vectors shift their distributional potential from west to east and south, which has implications for overall numbers of people exposed to these vector species. Although the total is reduced, malaria is likely to pose novel public health problems in areas where it has not previously been common.

## Background

Malaria is a vector-borne anthroponosis, transmitted in large part by *Anopheles *mosquitoes, that endangers more than 2.5 × 10^9 ^humans annually [1]. Its transmission cycle has been modeled in great detail [2,3], but application of such process-based models has generally been limited to local and regional scales [4,5], given challenges in spatially explicit parameter estimation. An alternative approach that offers broadest applicability is that of focusing on vector species' geographic distributions via ecological niche modeling techniques now well tested [6-8] within frameworks for reconstructing the geographic dimensions of disease transmission [9].

Implications of climate change for malaria transmission across Africa have been the subject of numerous commentaries [10-12] and a few attempted analyses [13-18]. One analysis [18] developed a global picture of climate change effects on malaria transmission, but relied on country-level classifications of risk, and did not discern the fine details of mosquito-species-specific distributional shifts. To date, then, specific climate change scenarios have yet to be applied to derivation of detailed projections of changing patterns of potential for malaria transmission based on mosquito vector species' distributions across Africa.

Ecological niche modeling uses nonrandom associations between known occurrences of species and digital geospatial data layers summarizing relevant environmental parameters to reconstruct the suite of environmental conditions under which the species is able to maintain populations without immigrational subsidy [19]. Modeled niches are generally conservative over considerable time periods [20,21], offering considerable predictive power regarding distributional ecology of species, including when environmental conditions are changing [8,22]. Previous studies have used these techniques to illuminate distributions of mosquito species under diverse circumstances [17,23-28]; perhaps most importantly, mosquito species' distributions are known to be highly sensitive to climatic variations, including on very short time scales [26]. Here, I integrate detailed climate change scenarios for the two most significant Africa-wide mosquito vector species based on ecological niche models and detailed spatial summaries of present-day human population distribution [29,30] to calculate shifting patterns of potential human exposure to malaria across Africa to be expected over the coming half-century.

## Methods

Occurrence data for vector species were derived from a recent detailed compilation [31] that was the basis of previous analyses [16,28]; in the present study, I focused on the two species with broad, continentwide distributions in the *Anopheles gambiae *complex: *A. gambiae sensu stricto *and *A. arabiensis*, for which 581 and 501 occurrence records were available, respectively. These data are based on sampling that was intense in some countries, and negligible or absent in others – as such, an extrapolative approach to their analysis becomes key. As such, I related the occurrence data to a suite of environmental data including dimensions of climate (annual mean temperature, mean monthly maximum temperature, mean monthly minimum temperature, annual precipitation; from [32]) and topography (slope, aspect, compound topographic index; from [33]). The relationship between the occurrence data and the environmental data sets – effectively the basis for the niche model – thus interpolates into unsampled regions based on the environmental characteristics of those regions, and thereby offers improved predictive ability regarding distributions. All environmental data sets were resampled to 0.1° spatial resolution for analysis.

Ecological niche models were generated using the Genetic Algorithm for Rule-Set Prediction (GARP) [34], a niche-modeling approach that has been the basis for most previous niche modeling applications to disease geography [9]. GARP is an evolutionary-computing method that builds ENMs based on non-random associations between known occurrence points for species and sets of GIS coverages describing the ecological landscape. Occurrence data are used as follows: 50% of occurrence data points are set aside for an independent test of model quality (extrinsic testing data), 25% are used for developing models (training data), and 25% are used for tests of model quality internal to GARP (intrinsic testing data). Distributional data are converted to raster layers by the GARP program; then, by random sampling from areas of known presence (training and intrinsic test data) and areas of 'pseudoabsence' (areas lacking known presences), two data sets are created, each of 1250 points; these data sets are used for rule generation and model testing, respectively.

The first rule is created by applying a method chosen randomly from a set of inferential tools (i.e., atomic rules that specify particular environmental value combinations as suitable, logistic regression, bioclimatic range rules, negated bioclimatic range rules [34]). The genetic algorithm consists of specially defined operators (e.g., crossover, mutation) that modify the initial rules, and thus the result are models that have "evolved" – after each modification, the quality of the rule is tested (to maximize both significance and predictive accuracy) and a size-limited set of best rules is retained. Because rules are tested based on independent data (intrinsic test data), performance values reflect the expected performance of the rule, an independent verification that gives a more reliable estimate of true rule performance. The final result is a set of rules that can be projected onto a map to produce a potential geographic distribution for the species under investigation.

Following recent recommendations [35], for each species, I developed 100 replicate random-walk GARP models, and filtered out 90% based on consideration of error statistics, as follows. The 'best subsets' methodology consists of an initial filter removing models that omit (omission error = predicting absence in areas of known presence) heavily based on the extrinsic testing data, and a second filter based on an index of commission error (= predicting presence in areas of known absence), in which models predicting very large and very small areas are removed from consideration. Specifically, in DesktopGARP, I used a "soft" omission threshold of 20%, and 50% retention based on commission considerations; the result was 10 'best subsets' models (binary raster data layers) that were summed to produce a best estimate of geographic prediction. I took as a prediction of suitable conditions those areas for which ≥8 of the 10 replicate models predicted potential for presence, and a prediction of unsuitable conditions those areas for which ≤4 of the 10 replicate models predicted potential for presence.

Future climate conditions were reconstructed based on two general circulation models (GCMs) that have been used to simulate future climates: those of the Hadley Centre (HadCM3) [36] and the Canadian Center (CGCM1) [37]. From each GCM, I analyzed two greenhouse gas emissions scenarios: the B2 scenario, which is a relatively conservative estimate of climate change, and the A2 scenario, which is more extreme in the climates reconstructed. As they are based on a 30 yr average around 2055, these models do not take into account potential effects of increased climate variability (El Niño events, in particular) on species' distributions. Because these future climate data are provided at a very coarse native spatial resolution (2.5 × 3.75°), I calculated expected changes in temperature (°C) and precipitation (mm) under each scenario from the relatively coarse raw model results; these expected changes were applied to the original IPCC current climate data layers to provide a final pixel resolution of ~30 × 30 km for future-climate data layers. Models developed based on present-day occurrence patterns and environmental variation were projected to these 4 views of likely future climate conditions.

Finally, I calculated human populations living within the potential distributions of these competent malaria vectors across sub-Saharan Africa based on the LandScan database [29,30], a 1 km resolution summary of present-day human population distributions globally. It should be noted that I make the explicit assumption of stability of human population distributions – although future population projections are available [38], they are overly coarse spatially to be greatly informative in this analysis, so this point remains as a future challenge for improvement. For each greenhouse gas emissions scenario, I averaged the results of the two climate models to yield single estimates of future potential distribution under that scenario. Intersecting the model predictions with the Landscan dataset, I calculated present-day numbers of people living in areas coinciding with potential distributional areas of each mosquito species, and present-day numbers of people living in areas converting from unsuitable to suitable or vice versa.

## Results

**Figure 1:**
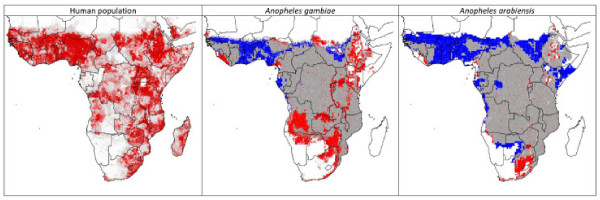
**Human distributions and modeled present and future malaria vector distributions in Africa**. Maps showing the basic panorama of human distribution across Africa, as well as modeled present and future distributions of the two major malaria vectors across Africa, *Anopheles gambiae *and *A. arabiensis*. Human population is shown as white – 0–25 persons, gray 26–441, pink 442–857, red 858–1273, dark red >1273. Mosquito species distributions: gray = areas modeled as suitable both at present and in future projections; blue = areas presently modeled as suitable, but that are not projected as suitable under future conditions; red = areas expected to become newly suitable for mosquito species under future conditions.

**Figure 2:**
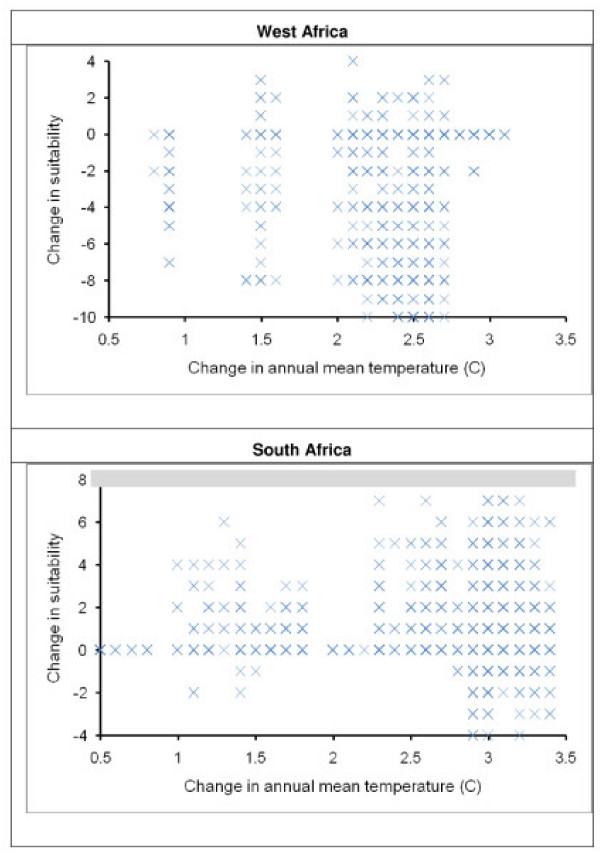
**Suitability changes in key regions**. Change in suitability (on a scale of -10 to +10, with +10 indicating maximum improvement of suitability between present and future) for *Anopheles gambiae *in West Africa and South Africa. In West Africa, the key conditions are for decline in suitability, whereas in South Africa, focus should be on increasing suitability.

*Anopheles gambiae *and *A. arabiensis*, the two most important malaria vectors in Africa, have broad distributions across Africa [28] (Fig. 1). I do not present detailed validations of model predictions across Africa in the present day because I had published such analyses in an earlier study based on precisely the same occurrence data set [28] – suffice it to say that model predictions were quite robust, offering confidence in the areas identified as suitable *versus *unsuitable. Projections of the ecological niche model onto likely future climate conditions indicated that both mosquito species are likely to see less suitable conditions for their populations across portions of West Africa, where temperature increases of 1.5–2.7°C are likely to be manifested (Fig. 2). In contrast, both species are likely to see improving conditions in regions of southern Africa, in areas where annual mean temperatures are increasing sufficiently to permit these species to establish populations (Fig. 2).

Present-day human populations coinciding spatially with the inferred geographic distributions of these vector species presently total 389,155,713 for *A. gambiae *and 520,289,130 for *A. arabiensis *over 10.5 × 10^6 ^and 14.5 × 10^6 ^km^2^, respectively. Under the two greenhouse gas scenarios, >30 million people are living in areas projected to see increasing exposure to *A. gambiae*, and >14 million people are living in areas projected to see increased exposure to *A. arabiensis*. At the same time, however, 78–111 million people are living in areas projected to see reduced exposure to *A. gambiae*, and 135–171 million people are living in areas projected to see reduced exposure to *A. arabiensis *(Table 1). These changing patterns are distributed unevenly across the continent: reduced exposure across West Africa and the Sahel, but increased potential for exposure in East Africa and southern Africa (Fig. 1).

**Table 1:** Human malaria vector exposure trends

Species	Greenhouse gas scenario	Area exposed (km^2^)	Human population explosed	Area no longer exposed (km^2^)	Human population no longer exposed
*Anopheles gambiae*	Present	10,508,761	389,155,713	10,508,761	389,155,713
	B2	+11.47%	+8.44%	-18.60%	-28.73%
	A2	+11.42%	+8.74%	-27.51%	-20.03%
					
*Anopheles arabiensis*	Present	14,528,957	520,289,130	14,528,957	520,289,130
	B2	+2.03%	+2.77%	-26.44%	-32.95%
	A2	+1.96%	+2.70%	-39.46%	-26.07%

Summary of overall tendencies in human exposure to two species of malaria vectors across Africa under two scenarios of greenhouse gas concentrations and consequent climate change, showing transitions from low-risk suitability categories to high-risk categories and vice versa, and the human population numbers that are associated.

## Discussion

This study illustrates the complexities of effects of changing climate conditions on spatial patterns of the potential for disease transmission. Ecological niche models were developed for the two most significant and broadly distributed malaria vectors in Africa and projected onto two greenhouse gas emissions scenarios for each of two global climate models. Clearly, a first priority is to expand these analyses from the two most broadly distributed species in the *Anopheles gambiae *complex to the broader suite of potential vectors across the continent, as, for instance, *A. funestus*, *A. nili*, and *A. moucheti *are competent vectors in Cameroun [39]. The present analysis was of necessity restricted to the *A. gambiae *complex for lack of access to high-quality, continentwide occurrence data for other species. A further limitation of this study is its analysis based on present-day human population levels and distributions, for lack of detailed information on future status; similarly, I neglect the effects of urbanization and interventions on mosquito vector distributions and malaria transmission rates.

In the previous global analysis [18], as in my more detailed analysis, increasing malaria risk was projected in East Africa and decreasing malaria risk was projected for West Africa, so the general pictures presented by the two analyses are parallel, but the detail offered in the present study is considerably greater. Nonetheless, my results require careful examination and exploration, because overall reductions in potential for human exposure nonetheless are achieved by reductions in some regions and increases in others.

Ecological niche models reconstruct patterns of association between species' occurrences and environmental variation across space and time [40], and offer a predictive understanding of distributional patterns that can extend over changing environmental conditions. They require testing and validation prior to use [41], however, which in the present case has been the subject of a detailed, published analysis demonstrating excellent predictivity of distributional patterns of the vector species even across broad, unsampled regions [28]. These tests, as well as more general validations of the analytical framework [7,42-44], increase confidence that projections of niche dimensions across periods of environmental change will also have predictive power. That mosquito species are likely to respond to changing climates is quite probable, particularly in light of recent studies demonstrating that mosquito distributions in time and space respond to the same sets of stimuli [26].

The climate change picture regarding shifting patterns of malaria vector distributions across Africa is complex. In general, future expectations are that, while fewer people live in areas that will be suitable for malaria vectors across West Africa, more people will be living in exposed areas in East Africa and southern Africa. Because West Africa is more densely populated than parts of eastern and southern Africa, a net decrease in potential for human exposure to these vectors results. For *A. gambiae*, 78–112 million fewer people are projected to be in suitable areas for the vector species, as compared with 33–34 million people living in areas becoming suitable; for *A. arabiensis*, reductions in potential for exposure are more dramatic: 136–171 million in areas no longer suitable, but only 14 million in areas newly suitable. The spatial distribution of these shifts, however, is perhaps the most intriguing result: malaria transmission risk is likely to shift eastward and southward, establishing new foci in areas presently lacking intense malaria transmission.

Adding still more complexity are comparisons of the two greenhouse gas emissions scenarios. The A2 scenario emphasizes a heterogeneous world, with regional self-reliance and preservation of local identities, which results in continuously increasing population, regional economic development and slower technological change. The B2 scenario, on the other hand, is one of local solutions to economic, social and environmental sustainability, global population increases at rates lower than A2. Curiously, though, the more extreme (in terms of climate change) A2 scenario is associated with 20–30% *less *human exposure to malaria across Africa, given its more extreme projected effects on *Anopheles *distributions, particularly in West Africa. The difference between the two scenarios is thus not so much in terms of how many people live in areas newly suitable for malaria vectors, but rather in terms of how many fewer people live in areas no longer suitable.

## Conclusion

Scientific results regarding implications of climate change are usually pessimistic in nature. This analysis departs somewhat from that theme – malaria vector species are expected to undergo distributional shifts in the face of changing climates that will leave fewer people overall in areas suitable for the important vector species. The net reduction nonetheless includes increasing malaria vector presence in areas of eastern and southern Africa that are not presently exposed to these species. As such, the picture is one of complex rearrangement of malaria transmission across Africa with climate change.

## Competing interests

The author declares that they have no competing interests.

## Authors' contributions

ATP developed entire manuscript

## Pre-publication history

The pre-publication history for this paper can be accessed here:

http://www.biomedcentral.com/1471-2334/9/59/prepub
